# Characterization of a novel yeast species *Metschnikowia persimmonesis* KCTC 12991BP (KIOM G15050 type strain) isolated from a medicinal plant, Korean persimmon calyx (*Diospyros kaki* Thumb)

**DOI:** 10.1186/s13568-017-0503-1

**Published:** 2017-11-10

**Authors:** Young Min Kang, Ji Eun Choi, Richard Komakech, Jeong Hwan Park, Dae Wook Kim, Kye Man Cho, Seung Mi Kang, Sang Haeng Choi, Kun Chul Song, Chung Min Ryu, Keun Chul Lee, Jung-Sook Lee

**Affiliations:** 1 Korea Institute of Oriental Medicine (KIOM), 1672 Yuseong-daero, Yuseong-gu, Daejeon, 34054 Republic of Korea; 2 University of Science & Technology (UST), Korean Medicine Life Science, Campus of KIOM, 1672 Yuseong-daero, Yuseong-gu, Daejeon, 34054 Republic of Korea; 3 Natural Chemotherapeutics Research Institute (NCRI), Ministry of Health, P.O. Box 4864, Kampala, Uganda; 4 Bioresources Industrialization Research Division, Nakdonggang National Institute of Biological Resources, Donam 2-gil 137, Sangju-si, Gyeongsangbuk-do 37242 Republic of Korea; 5 Department of Food Science, Institute of Fusion Biotechnology, Gyeongnam National University of Science and Technology, Jinju, 660-758 Republic of Korea; 6 Forest Research Department, Gyeongsangnam-do Forest Environment Research Institute, Jingu, 52615 Republic of Korea; 7 AtoGen Co., Ltd., 11-8, Techno 1-ro, Yuseong-gu, Daejeon, 34015 Republic of Korea; 8 Korea Research Institute of Bioscience and Biotechnology (KRIBB), Daejeon, Republic of Korea; 9 Department of Food Science and Biotechnology, Chungbuk National University, Cheongju, 361-763 Republic of Korea

**Keywords:** *Diospyros kaki* Thumb, Persimmon, Korean herbal medicine, Yeast, *Metschnikowia persimmonesis* sp. nov

## Abstract

The yeast strain *Metschnikowia persimmonesis* Kang and Choi et al., sp. nov. [type strain KIOM_G15050 = Korean Collection for Type Cultures (KCTC) 12991BP] was isolated from the stalk of native persimmon cultivars (*Diospyros kaki* Thumb) obtained from different regions of South Korea and was characterized phenotypically, genetically, and physiologically. The isolate grew between 4 and 40 °C (optimum temperature: 24–28 °C), pH 3–8 (pH optimum = 6.0), and in 0–4% NaCl solution (with optimal growth in absence of NaCl). It also exhibited strong antibiotic and antimicrobial activities. Morphologically, cells were characterized by the presence of long, needle-shaped ascospores. Based on 18S ribosomal DNA gene sequence analysis, the new species was found to belong to the genus *Metschnikowia* as a sister clade of *Metschnikowia fructicola.* We therefore conclude that this yeast isolate from *D. kaki* is a new member of the genus *Metschnikowia* and propose the name *M. persimmonesis* sp. nov. This strain has been deposited in the KCTC for future reference. This discovery provides a basis for future research on *M. persimmonesis* sp. nov., including its possible contribution to the medicinal properties of the host persimmon plant.

## Introduction

The genus *Metschnikowia* of the family *Metschnikowiaceae* (Order *Saccharromycetales*) comprises single-celled species that reproduce via budding of vegetative cells and is characterized by the presence of one or two needle-shaped ascospores on elongated asci (Mendonca-Hagler et al. 1993; Pretorius 2000; Marinoni et al. 2006; Kuan et al. 2016). A total of 35 *Metschnikowia* species have been described to date in a wide range of hosts, including flowers, fruits, flower-pollinating insects, and lacewings (Mendonca-Hagler et al. 1993; Lachance et al. 2005; Guzmán et al. 2013; De Vega et al. 2014; Kuan et al. 2016; Álvarez-Pérez et al. 2016). Yeasts provide a variety of unique bioactive metabolites that have medicinal importance (Mager and Winderickx 2005; VanderMolen et al. 2013). They are also used as biocontrol agents along with bacteria in agriculture, especially for the prevention of post-harvest diseases in fruits (Janisiewicz et al. 2001; Kurtzman and Droby 2002; Spadaro et al. 2008; Csutak et al. 2013; Türkel et al. 2014) such as *Metschnikowia fructicola* isolated from grapes (Kurtzman and Droby 2002). Persimmon (*Diospyros kaki* Thumb.); a Korean medicinal plant (Mallavadhani et al. 1998; Kotani et al. 2000; Heo 2001; Giordani et al. 2011; Tsubaki et al. 2012) is one of the many plants that are inhabited by a number of fungal species, including the microfungus *Fusicladium levieri* (Scholler 2003); *Metschnikowia reukaufii* and *M. gruessii* which are mainly found in the nectar (Canto et al. 2015). In a study, it was observed that *D. kaki* fruit stalk is inhibited by a number of microorganisms including bacteria and fungi (Choi et al. 2017). However, despite the fact that a number of studies have attempted to isolate yeast inhabiting different parts of persimmon over the years, no study is known to have been conducted to isolate yeast species from the fruit stalk of *D. kaki.* In this study therefore, we describe a new yeast species *Metschnikowia persimmonesis* Kang and Choi et al., sp. nov. (type strain KIOM_G15050 = KCTC 12991BP) isolated from the stalk of native Korean *D. kaki* cultivars obtained from different regions of South Korea. *M. persimmonesis* was identified as a novel species based on pyrosequencing of its 18S rDNA gene. We characterized this species morphologically, genetically, and physiologically, and evaluated its growth in the presence of various nutrients and environmental stimuli. We found that *M. persimmonesis* produces needle-shaped ascospores which is a basic characteristic of members of the genus *Metschnikowia.* Its taxonomic membership was confirmed by a phylogenetic analysis that revealed *M. fructiola* as the closest sister species. This novel yeast strain has been given Korean Patent Submission Number (10-2016-0137873) and international Patent Submission Number (PCT-KR2017-010681) and deposited in the Korean Collection for Type Cultures (KCTC) for future reference and research.

## Materials and methods

### Sample preparation and specimen isolation

**Fig. 1:**
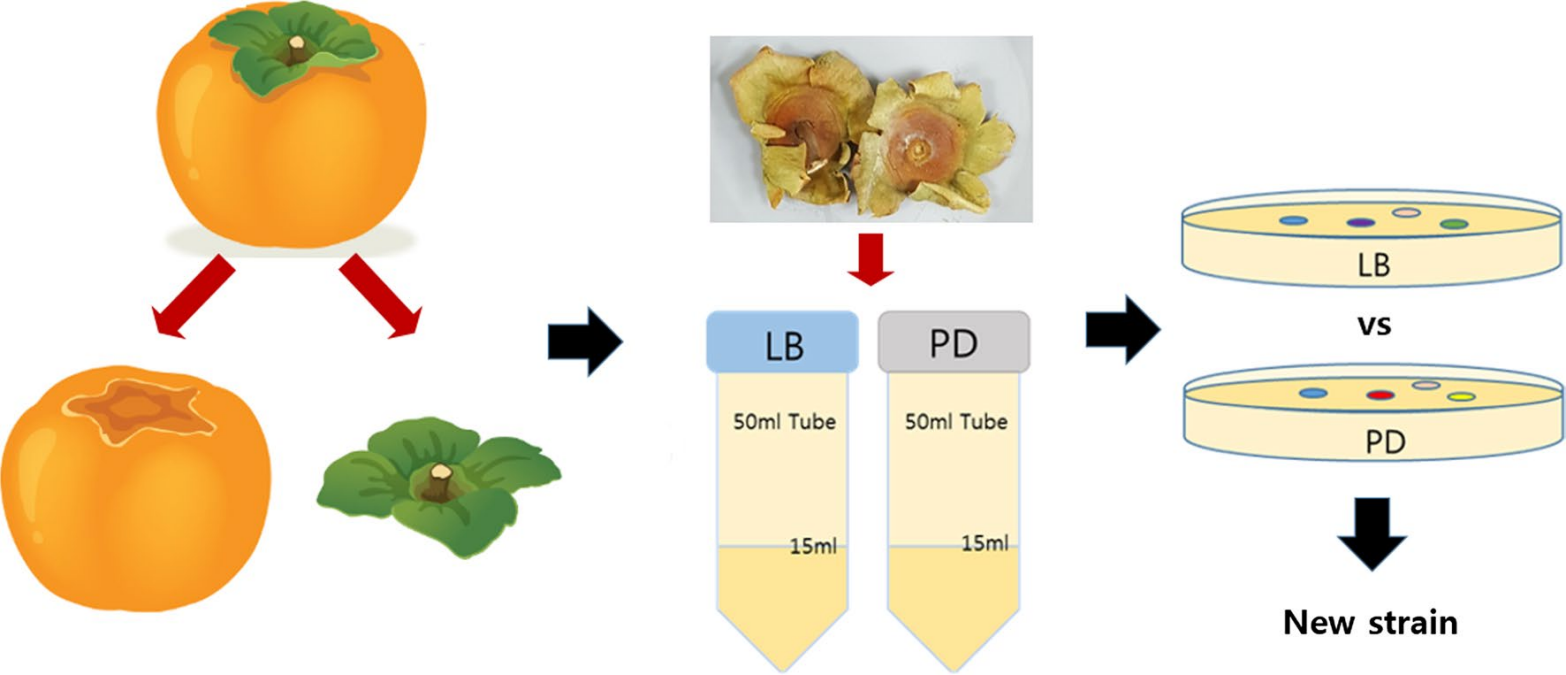
Steps in preparing *D. kaki* samples

The origin of the yeast strain used in this study was the fruit stalk and calyx of *D. kaki* obtained in South Korea and processed by suspending the cleaned dried and pulverized samples in 50 ml Luria-Bertani (LB) or potato dextrose (PD) medium (Fig. 1). A 15-ml volume of each sample from each of the medium was transferred to a 50-ml tube and incubated at 25 °C with shaking at 100 rpm for 48 h. LB and PD cultures were streaked onto agar-containing LB and PD medium respectively and a single colony was isolated by sub-culturing four times via streaking on fresh plates. Single colonies were stored at − 20 °C in a 1.5-ml Eppendorf tube.

### Substrate culture and strain characterization

Colonies growing on potato dextrose agar (PDA) and LB agar medium were inoculated in 5 ml LB liquid medium or potato dextrose broth (PDB) in sterilized test tubes and cultured at 28 °C. To obtain growth curves, 100 ml LB liquid medium or PDB were incubated at 28 °C with shaking at 150 rpm in a 500-ml round flask. The inoculum was combined with a 3% volume of culture medium containing preculture (with 595-nm absorbance values of 0.379 and 0.377, respectively), and cultured for 0, 6, 12, 18, 24, 36, 48, 72, 96, 120, and 168 h. Absorbance readings at 595 nm were obtained for tenfold dilutions of sample and used to generate a standard curve; this was used to determine the number of viable cells, which is expressed as colony-forming units (cfu) per μl. The number of cells in the culture was calculated by counting the viable cells as follows: 1 ml of culture was transferred to a test tube containing 9 ml sterilized water and serially diluted. Three replicates of 100 cells each were prepared on separate plates that were incubated at 28 °C for 2 days. The number of colonies on each plate was counted and is expressed as an average value (cfu per ml). *M. persimmonesis* was also sub-cultured for 24 h in tryptic soy agar (TSA), nutrient agar (NA), R2A, Bennett’s agar, and ISP2.

### Microscopic examination of *M. persimmonesis*

The yeast strain was spread on PDA plates using sterile bent streaking rod. Plates were then incubated at 28 °C for 48 h. Suspected colonies of *M. persimmonesis* yeast were re-streaked on sterile PDA plates and incubated at 28 °C for 48 h. This procedure was repeated three times to obtain pure colonies of *M. persimmonesis* which was then examined by microscopy. A wet mount slide was prepared for examination in which a drop of water was placed on a microscope slide and a small amount of yeast cells were transferred to the drop with a sterile streaking stick and stirred. The drop was covered with a coverslip and viewed under the microscope. Cell morphology was evaluated by phase-contrast microscopy (Eclipse 80i; Nikon, Tokyo, Japan).

Mating compatibility of the isolated colonies was performed by culturing on yeast carbon base (Difco) supplemented with 1.5% agar and 0.01% ammonium sulfate at 28 °C then periodically examined by phase contrast microscopy (Eclipse 80i; Nikon, Tokyo, Japan).

### *M. persimmonesis* DNA extraction and quantification

Single colonies from the 13 samples cultured in LB and PD were labeled as L and P, respectively; 100 mg of each sample were pulverized and combined with 200 μl distilled water. The mixture was heated at 100 °C for 10 min. Genomic DNA was extracted using a DNeasy Plant Mini kit (Qiagen, Hilden, Germany). The amount and quality of genomic DNA were verified by electrophoresis on a 0.8% agarose gel, and the concentration was determined at 260 nm using a Nano Drop 1000 spectrophotometer (Nano Drop Technologies, Wilmington, DE, USA).

### Polymerase chain reaction (PCR) amplification and identification of *M. persimmonesis* based on 18S rDNA sequence

Polymerase chain reaction amplification of 18S rDNA was performed in a final reaction volume of 30 μl using 30 pmol of the universal solvent-based primers ITS1 (5′-TCC GTA GGT GAA CCT GCG G-3′; sequence ID: 3) and ITS4 (5′-TCC TCC GCT TAT TGA TAT GC-3′; sequence ID: 4), 20 ng genomic DNA, and Solg 2× Taq PCR Pre-Mix (Solgent, Daejeon, Korea). The reaction was carried out in a 9700 thermal cycler (Applied Biosystems, Singapore) under the following conditions: 95 °C for 15 min; 35 cycles of 50 °C for 40 s, 50 °C for 40 s, and 72 °C for 90 s; and 72 °C for 5 min. A 10-μl volume of the amplified 18S rDNA product was visualized by 1.5% agarose gel electrophoresis and was observed as a 600-bp band under ultraviolet light. The DNA fragment was purified using a kit (Solgent) and inserted into the pGEM-Teasy vector (Promega, Madison, WI, USA), which was transformed into XL1-Blue MRF competent cells (Stratagene, La Jolla, CA, USA). Single colonies were selected for insert verification. T7 and SP6 primer sites were analyzed with an ABI3730 automatic DNA sequencer (Applied Biosystems, Foster City, CA, USA) and compared with sequences in the National Center for Biotechnology Information (NCBI) Genbank database (http://www.ncbi.nlm.nih.gov). The GenBank Accession Number for the 18s rDNA and 26s rDNA of the yeast *M. persimmonesis* was MF446617 and MF446618 respectively.

### DNA extraction, PCR, and pyrosequencing

Pyrosequencing methods have been described in detail elsewhere (Buée et al. 2009; Serkebaeva et al. 2013). Colonies on LB and PD agar plates were collected using sterile streaking sticks and transferred to sterile plastic conical tubes for identification by pyrosequencing. Samples were ground using a Precellys Grinder (Bertin Technologies, Montigny-le-Bretonneux, France), centrifuged, and washed twice with 1× phosphate-buffered saline (Biowhittaker, Walkersville, MD, USA). Pellets were stored at − 20 °C in 1.5-ml Eppendorf tubes before DNA isolation.

Genomic DNA was extracted from colonies of 13 samples using the MagListo 5M Genomic DNA Extraction kit (Bioneer, Daejeon, Korea) according to the manufacturer’s instructions. The DNA concentration was determined at 220 nm on a NanoDrop 1000 spectrophotometer and sample quality was confirmed on a 1% agarose gel. The 600-bp 18S DNA gene was amplified using the universal fungal 18S DNA primers ITS1 and ITS4. PCR amplification was carried out in a 2720 thermal cycler (Applied Biosystems, Foster City, CA, USA) under the following conditions: 95 °C for 15 min; 35 cycles of 95 °C for 20 s, 53 °C for 40 s, and 72 °C for 90 s; and 72 °C for 5 min.

### Classification and identification of *M. persimmonesis*

Purified PCR products were used to construct gene libraries and determine nucleotide sequences using a GS Junior Titanium Sequencing kit (Roche Diagnostics, Indianapolis, IN, USA). Sequence reads obtained by pyrosequencing were analyzed as previously described (Kim et al. 2012; Diouf et al. 2015). Sequences obtained with GS Junior were compared with the 18S rDNA gene sequences from the non-redundant sequence database using the NCBI Basic Local Alignment Search Tool (BLAST; v.2.2.30+) for taxonomic identification and analysis of microbial diversity. Each sequence was then classified taxonomically using NCBI Metagenome Analyzer (MEGAN; v.5.10.1), and the results were used for taxonomic classification. Isolates were classified according to the hierarchy of super-kingdom, kingdom, phylum, class, order, family, genus, and species. A rooted phylogenetic tree was generated by assigning all sequences to each node. Each group of identified microorganisms is presented according to the Integrated Taxonomic Information System (ITIS) taxonomic counting heat maps. Microsoft Excel (Redmond, WA, USA) was used for data analysis.

### Growth characteristics of *M. persimmonesis* at various pH

Solutions of sterilized tryptic soy broth (TSB) medium (20 ml) with various pH were prepared and sterilized with a 0.2-μm pore filter. Culture broth was collected in TSA medium and resuspended in 3 ml TSB. A 100-μl volume of each suspension was inoculated and incubated at 28 °C for 2 days with shaking at 150 rpm. Cell viability was determined by measuring the turbidity at 595 nm with a spectrophotometer.

### Growth characteristics of *M. persimmonesis* at various NaCl concentrations

The growth characteristics of isolates were evaluated in nutrient broth (NB; 20 ml) containing NaCl concentrations of 0, 0.1, 0.3, 0.5, 1, 2, 3, 4, 5, 7, 10, 12, 15, 18, 20, and 24% (with Ml). Cultures were grown on nutrient agar (NA) and then resuspended in 3 ml NB; 100 μl of culture were then incubated at 28 °C with shaking at 150 rpm for 2 days. Yeast growth was then determined by measuring the turbidity of cells at 595 nm with a spectrophotometer.

### Growth characteristics of *M. persimmonesis* at various temperatures

Isolates were subcultured in TSA; cultures were transferred to 125-ml Erlenmeyer flasks containing 30 ml of TSB and incubated at temperatures of 4, 10, 15, 18, 20, 24, 28, 30, 35, 37, 40, 45, and 50 °C on a shaker at 160 rpm. Growth in TSB medium was evaluated by measuring turbidity at 640 nm at predetermined time intervals over 48 h at the above temperatures, as previously described (Janisiewicz et al. 2001).

### Saccharide test

API 20 C AUX and API 5 CH (both from bioMérieux, Marcy l’Étoile, France) were used to identify strains for the sugar (carbon) availability test according to the manufacturer’s protocols. These tests include 19 carbohydrate assimilation test strains along with a negative control and are based on turbidity measurements. Isolates were cultured on PDA and TSA plates. Colonies were collected and re-suspended in 0.85% NaCl to obtain a MacFarland turbidity of 2. A 100-μl aliquot of the turbid solution was thoroughly mixed with 7 ml API 20 C medium ampoule 2, and the resultant mixture was transferred to API 20 C AUX and API 5 CH cups using a sterile pipette. Strips were read after incubation for 48 h at 30 °C.

### Antimicrobial activity (inhibition) test

The disc-diffusion method (Balouiri et al. 2016) was used to evaluate antimicrobial activity of the isolates. Agar plates were inoculated with a standardized inoculum of *Botrytis cinerea* as the test microorganism. Filter paper discs containing predetermined concentrations of *M. persimmonesis* were placed on the agar surface. The plates were then incubated at 26 °C for 72 h, after which the diameter of growth inhibition zones was measured. The procedure was repeated using *Fusarium oxysporum* as the test organism instead of *B. cinerea.*

### Antibiotic test

Antibiotic susceptibility was evaluated using 6-mm BBL Sensi-Discs (BD Biosciences, Franklin Lakes, NJ, USA) containing nystatin (50 μg), nystatin (30 μg), salinomycin (30 μg), amphotericin (2 μg), or gramicidin S (30 μg) and other antibiotics including teicoplanin, amikacin, ampicillin, lincomycin, nalidixic acid, kanamycin, gentamicin, streptomycin, neomycin, penicillin, vancomycin, erythromycin, oleandomycin, amoxicillin, spiramycin, rifampicin, polymixin B, bacitracin, tetracycline, cycloheximide, roxithromycin, sphingomyelin, apramycin, hygromycin B, capreomycin, sisomycin, phosphomycin, chloramphenicol, and streptomycin.

## Results

### Morphology

**Fig. 2:**
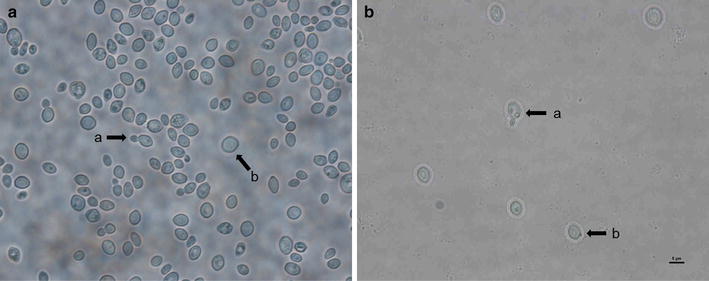
*Metschnikowia persimmonesis* KIOM_G15050 (KCTC 12991BP)

After 3 days of culture at 28 °C on PDA, cells were globose (3.0−7.0 × 4.0–9.0 µm) or ovoidal (6.0–9.0 µm) and grew as single cells or in small clusters (Fig. 2a). Budding cells and chlamydospores were common (Fig. 2a, b). Colonies were round, convex, smooth, and red-pigmented white.

### Growth curves characteristics of *M. persimmonesis* in PDB and LB medium

**Fig. 3:**
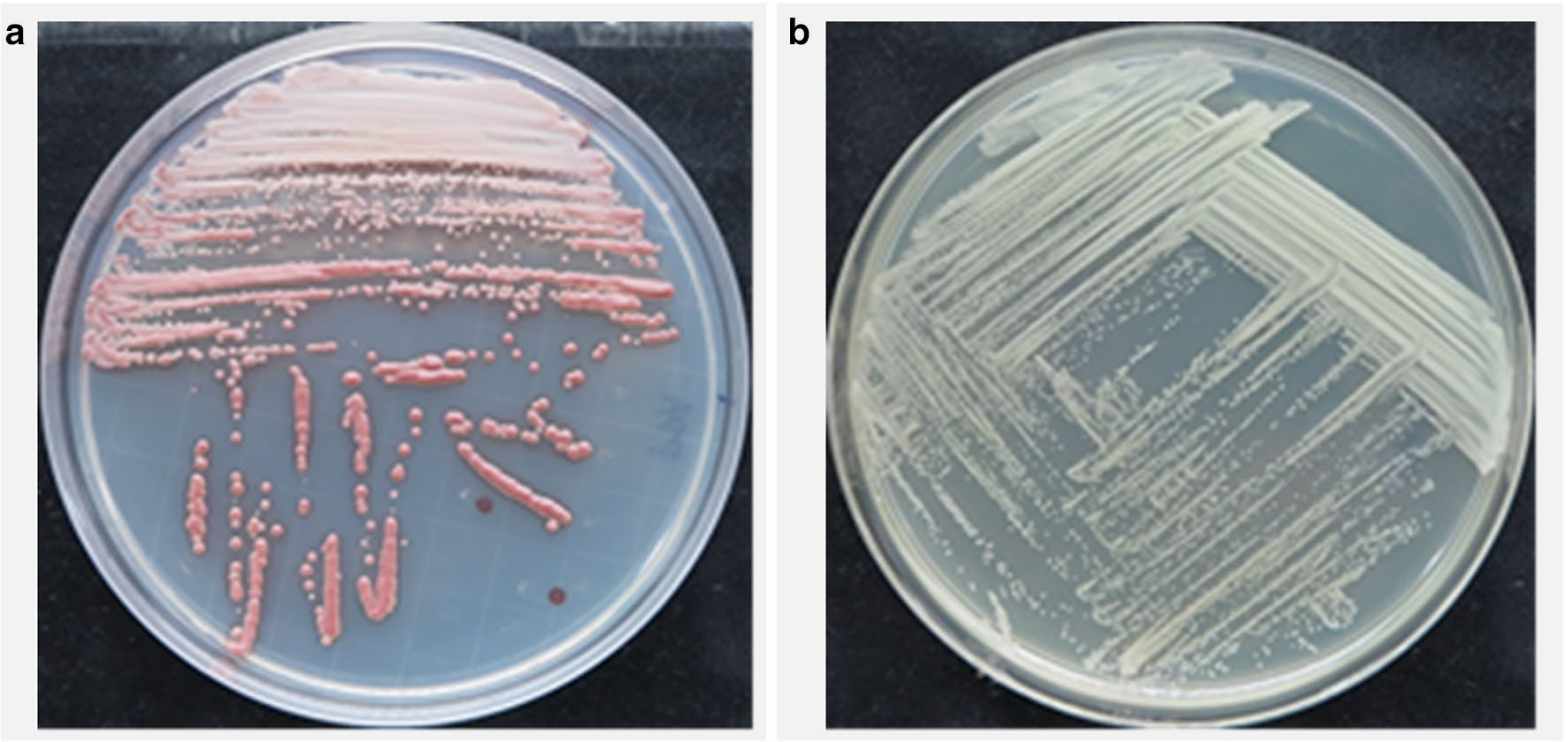
Growth of *M. persimmonesis* on different media

*Metschnikowia persimmonesis* that grew in PDA and TSA media (Fig. 3) was used for growth curve generation and strain characterization.

**Fig. 4:**
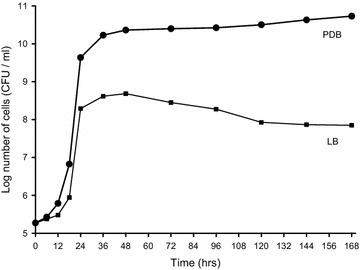
Growth according to medium of *Metschnikowia persimmonesis* KIOM G15050 strain

*Metschnikowia persimmonesis* grew rapidly from 12 to 24 h after induction in PDB (Fig. 4). The growth rate subsequently slowed between 36 and 168 h and then increased slightly to reach a maximal value at 168 h. A similar trend was observed in LB medium, except that growth was maximal at 48 h and then decreased up until the end of the culture period at 168 h.

### Standard growth curve for *M. persimmonesis*

**Fig. 5:**
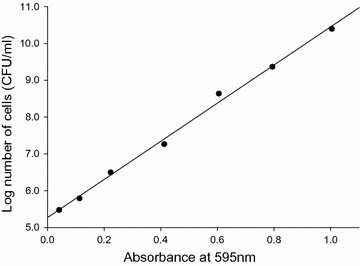
Bacterium amount standard curve of *Metschnikowia persimmonesis* KIOM G15050 strain

Samples cultured for 2 days were diluted to predetermined ratios and the number of cells was counted for samples with an absorbance of 1.0 or less to prepare a standard curve (Fig. 5). The equation of the line for the standard curve was determined as follows.

$${\text{Y }} = { 5}. 2 7 { } + { 5}. 1 8 {\text{x }}({\text{x:}}{\text{ absorbance}},{\text{ y:}}{\text{ Log no}}.{\text{ {of cells}}})$$

### Growth characteristics of *M. persimmonesis* at different pH

**Fig. 6:**
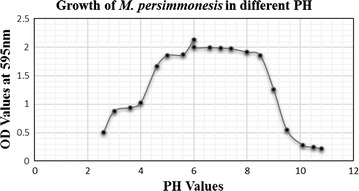
Growth of *M. persimmonesis* in different pH range

The growth of *M. persimmonesis* in TSB varied from pH 2.6 to 10.6. The growth rate increased with increases in pH up to pH 6.0 before declining (Table 1 and Fig. 6).

**Table 1:** Summary of growth tests and other test characteristics of *M. persimmonesis*

Tests	Results	Tests	Results	Tests	Results	Tests	Results	Tests	Results
Assimilation at 96 h of:		Oxidation at 96 h of:		Antimicrobial susceptibility test		API 2 °C AUX test		API 50 CH test	
Water	−	Water	−	Teicoplanin	*−	Glucose	+	Control	−
Fumaric acid	v	Acetic acid	v	Amikacin	*−	Glycerol	+	Glycerol	+
l-Malic acid	v	Formic acid	−	Ampicillin	*−	2-Keto-d-gluconate	+	Erythritol	−
Succinic acid mono-methyl ester	−	Propionic acid	−	Lincomycin	*−	l-Arabinose	–	d-Arabinose	−
Bromo-succinic acid	−	Succinic acid	+	Nalidixic acid	*−	d-Xylose	+	l-Arabinose	−
l-Glutamic acid	v	Succinic acid mono-methyl ester	v	Kanamycin	*−	Adonitol	+	d-Ribose	−
γ-Amino-butyric acid	−	l-Aspartic acid	−	Gentamicin	*−	Xylitol	+	d-Xylose	+
α-Keto-glutaric acid	−	l-Glutamic acid	v	Streptomycin	*−	d-Galactose	+	l-Xylose	−
2-Keto-d-gluconic acid	v	l-Proline	v	Neomycin	*−	Inositol	−	d-Adonitol	+
d-Gluconic acid	v	d-Gluconic acid	+	Penicillin	*−	d-Sorbitol	^+^	Methyl-*β*-d-xylopyranoside	−
Dextrin	−	Dextrin	−	Vancomycin	*−	α-Methyl-d-glucoside	^+^	d-Galactose	+
Inulin	−	Inulin	−	Erythromycin	*−	N-Acetyl-d-glucosamine	+	Glucose	+
d-Cellobiose	+	Cellobiose	+	Oleandomycin	*−	d-Cellobiose	+	d-Fructose	+
Gentiobiose	v	Genntiobiose	+	Amoxicillin	*−	d-Lactose(bovine origin)	−	d-Mannose	+
Maltose	−	Maltose	+	Spiramycin	*−	d-Maltose	^+^	l-Sorbose	+
Maltotriose	−	Maltotriose	+	Rifampicin	*−	d-Saccharose (Sucrose)	^+^	l-Rhamnose	−
d-Melezitose	v	d-Melezitose	+	Polymixin B	*−	d-Trehalose	−	Dulcitol	−
d-Melibiose	−	d-Melibiose	−	Nystatin	*+	d-Melezitose	^+^	Inositol	−
Palatinose	−	Palatinose	+	Bacitracin	*−	d-Raffinose	−	d-Manntol	+
d-Raffinose	−	d-Raffinose	−	Tetracycline	*−			d-Sorbitol	+
Stachyose	−	Stachyose	−	Cycloheximide	*+			Methyl-*α*-d-mannopyranoside	−
Sucrose	−	Sucrose	+	Roxithromycin	*−			Methyl-*α*-d-glucoopyranoside	+
d-Trehalose	v	d-Trehalose	−	Sphingomyelin	*−			N-Aceylglucosamine	+
Turanose	v	Turanose	+	Apramycin	*−			Amygdalin	+
N-Acetyl-d-glucosamine	−	N-Acetyl-d-glucosamine	+	Salinomycin	*+			Arbutin	+
d-Glucosamine	v	α-d-Glucose	+	Hygromycin B	*−			Esculin	+
α-d-Glucose	+	d-Galactose	v	Capreomycin	*−			Salicin	+
d-Galactose	v	d-Psicose	−	Sisomycin	*−			d-Celoibiose	+
d-Psicose	−	l-Sorbose	+	Amphotericin	*+			d-Maltose	+
l-Rhamnose	−	Salicin	v	Gramicidin S	*+			d-Lactose (bovine origin)	−
l-Sorbose	−	d-Mannitol	+	Phosphomycin	*−			d-Melibiose	−
α-Methyl-d-glucoside	v	d-Sorbitol	+	Chloramphenicol	*−			d-Saccharose (sucrose)	+
β-Methyl-d-glucoside	v	d-Arabitol	−	Streptomycin	*−			d-Trehalose	+
Amygdalin	−	Xylitol	v					Inulin	−
Arbutin	v	Glycerol	−					d-Melezitose	+
Salicin	v	Tween 80	−					d-Rafinose	−
Maltitol	v							Amidon (starch)	−
d-Mannitol	v							Glycogen	−
d-Sorbitol	−							Xylitol	+
Adonitol	v							Gentiobiose	+
d-Arabitol	−							d-Turanose	+
Xylitol	v							d-Lyxose	−
i-Erythritol	−							d-Tagatose	−
Glycerol	v							d-Fucose	−
Tween 80	−							l-Fucose	−
l-Arabinose	−							d-Arabitol	+
d-Arabinose	−							l-Arabitol	−
d-Ribose	−							Potassium glucoaate	+
d-Xylose	+							Potassium 2-ketogluconate	+
Succinic acid mono-methyl ester plus d-xylose	v							Potassium 5-ketogluconate	−
N-Acetyl-l-glutamic acid plus d-xylose	v								
Quinic acid plus d-xylose	+								
d-Glucuronic acid plus d-xylose	v								
Dextrin plus d-xylose	v								
α-d-Lactose plus d-xylose	v								
d-Melibiose plus d-xylose	v								
d-Galactose plus d-xylose	+								
m-Inositol plus d-xylose	v								
1,2- Propanediol plus d-xylose	v								
Acetoin plus d-xylose	v								

V , variable; −, negative; **+,** positive; *+, sensitive or susceptible to the antibiotic; *−, resistant to the antibiotic

### Growth characteristics of *M. persimmonesis* at different NaCl concentrations

**Fig. 7:**
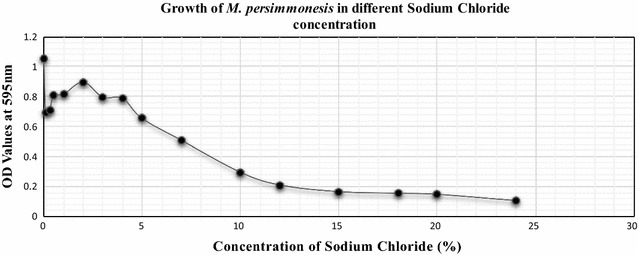
Growth of *M. persimmonesis* in different sodium chloride concentration

*Metschnikowia persimmonesis* was cultured in nutrient broth with different NaCl concentrations. *M. persimmonesis* exhibited maximal growth in the absence of NaCl; the growth rate was inversely related to NaCl concentration, with the lowest rate recorded at 24% NaCl (Fig. 7).

### Growth temperature

*Metschnikowia persimmonesis* was inoculated on TSA and cultured at temperatures ranging from 4 to 50 °C. Growth was inhibited at higher temperatures (45 and 50 °C) and the optimum growth temperature was 25 °C.

### Physiological tests

After incubation for 24–48 h, *M. persimmonesis* cultured on PDA and TSA was tested for carbon source availability using API 20 C AUX and API 5 CH. *M. persimmonesis* was found to metabolize glycerol, d-xylose, d-adonitol, d-galactose, glucose, d-fructose, d-mannose, mannose, l-sorbose, d-mannitol, d-sorbitol, methyl-α-d-glucopyranoside, glucopyranoside, *N*-acetylglucosamine, amygdalin, arbutin, esculin, salicin, d-cellobiose, d-maltose, d-saccharose (sucrose), d-trehalose, d-melezitose, xylitol, and zenose. Carbohydrates added as a carbon source included gentiobiose, d-turanose, d-arabitol, potassium gluconate, and potassium 2-ketogluconate. However, erythritol, d-arabinose, l-arabinose, d-ribose, l-xylose, d-xylopyranoside, l-rhamnose, dulcitol, inositol, d-methyl-α-d-mannopyranoside, d-lactose (bovine origin), d-melibiose, insulin, d-raffinose (d-lactose, amidon (starch), glycogen, d-lyxose, d-tagatose, d-fucose), l-fucose, l-arabitol, and potassium 5-ketogluconate carbohydrates were not used as carbon sources. We also found that d-sorbitol, α-methyl-d-glucoside, d-maltose, d-saccharose (sucrose), and d-melezitose were used as a carbon source and induced pigment production (Table 1).

## Discussion

**Fig. 8:**
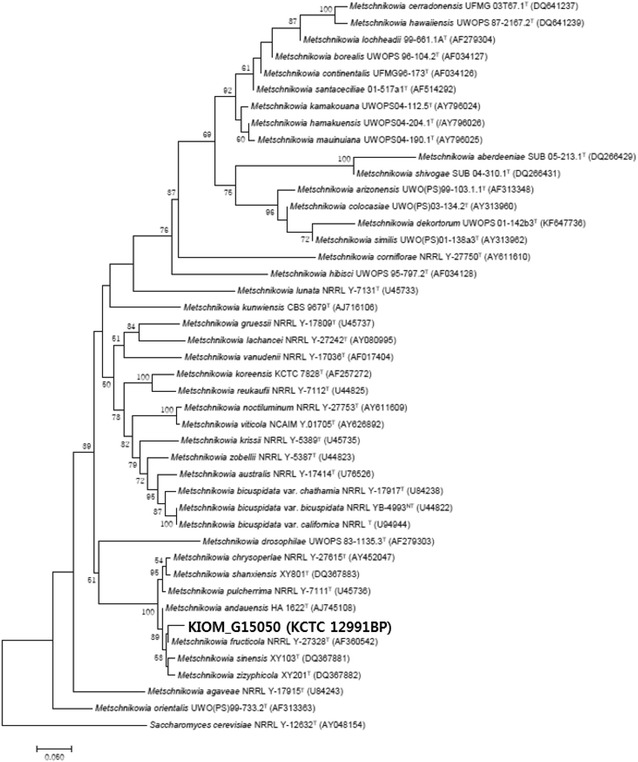
Phylogenic tree indicating position of *M. persimmonesis* in genus *Metschnikowia*

*Metschnikowia* represents one of the most phylogenetically diverse genera of ascomycetous yeast comprising of 35 known species in a wide range of habitats (Kuan et al. 2016), and yet are difficult to distinguish due to similarities in assimilation reactions among different species (Kurtzman and Droby 2002). Both *M. persimmonesis* and *M. fructicola* show very similar assimilation reactions, apart from the fact that they exhibit negative and positive reactions, respectively, to l-sorbose, sucrose, and maltose (Table 1). A microscopic examination revealed that *M. persimmonesis* has unique morphology, including a thick wall and elongated asci (Fig. 2). But since these characteristics are also shared by *M. fructicola* (Kurtzman and Droby 2002), the growth test and morphological features alone cannot be used to distinguish *M. persimmonesis* from *M. fructicola.* The use of phylogenetic analysis of conserved DNA and protein sequences is vital in the taxonomical classification (Cummings et al. 2013). In fact, recent study showed that pyrosequencing makes it possible to simultaneously profile large numbers of samples (Chun et al. 2010) using sequence repeats of the chromosomal rDNA array. Indeed, rDNA repeats within a genome have identical sequences due to sequence homogenization, and can therefore be used for species identification and barcoding (Sipiczki et al. 2013). The compared sequences generated by GS Junior to the NCBI nucleotide sequence database using BLAST gave a resultant file that was saved in xml format, and taxonomic classification was performed using NCBI taxonomic information and MEGAN v.5.10.6. More than 660,000 taxon types were returned by the search, with individual species classified in a hierarchical manner (super kingdom, kingdom, phylum, class, order, family, genus, and species). MEGAN provided a lowest common ancestor assignment algorithm that was used to assign the lowest taxon to each sequence. All sequences were assigned to each node to complete the rooted phylogenetic tree. The generated IT IS taxonomic counting heat maps were used to complete the identification of *M. persimmonesis* and the entries in Genbank placed this strain of yeast as a sister clade of *M. fructicola* (Fig. 8).

The maximum growth rate of *M. persimmonesis* in TSB was recorded at pH 6.0 (Table 1 and Fig. 7). This is within the same pH range for other species within this genus such as *M. andauensis*, which showed maximal growth at pH 6.5 (Manso et al. 2014) and *M. pulcherrima*, which showed maximum growth rate at optimum pH ranging from 5.0 to 7.5 (Spadaro et al. 2010). These results therefore indicate that the optimal pH for growth of *Metschnikowia* species is between pH 5–7.5.

The decrease in growth of *M. persimmonesis* with increase in Sodium chloride concentration is related to previous studies on some members of genus *Metschnikowia* in which the growth of *M. arizonensis* and *M. dekortorum* on yeast mold agar with 5% NaCl was weakly positive and was inhibited at a just a concentration of 10% (Lachance and Bowles 2002). Indeed, yeast growth decreases with increasing NaCl concentration (Speakman et al. 1928). Thus, growth inhibition of *M. persimmonesis* at 24% NaCl concentration (Fig. 8) further proves that low tolerance to NaCl may be a general characteristic of *Metschnikowia* species.

The optimum growth temperature (25 °C) for *M. persimmonesis* is within the same optimum growth temperature range for some of the members of genus *Metschnikowia.* For instance, an acid protease from the marine yeast *M. reukaufii* W6b exhibited maximal activity at 25 °C (Li et al. 2010), which was the temperature that promoted optimum growth of *M. pulcherrima* (Csutak et al. 2007). In fact, most yeasts have optimal growth temperatures between 20 and 25 °C (Kurtzman and Fell 1998).

**Fig. 9:**
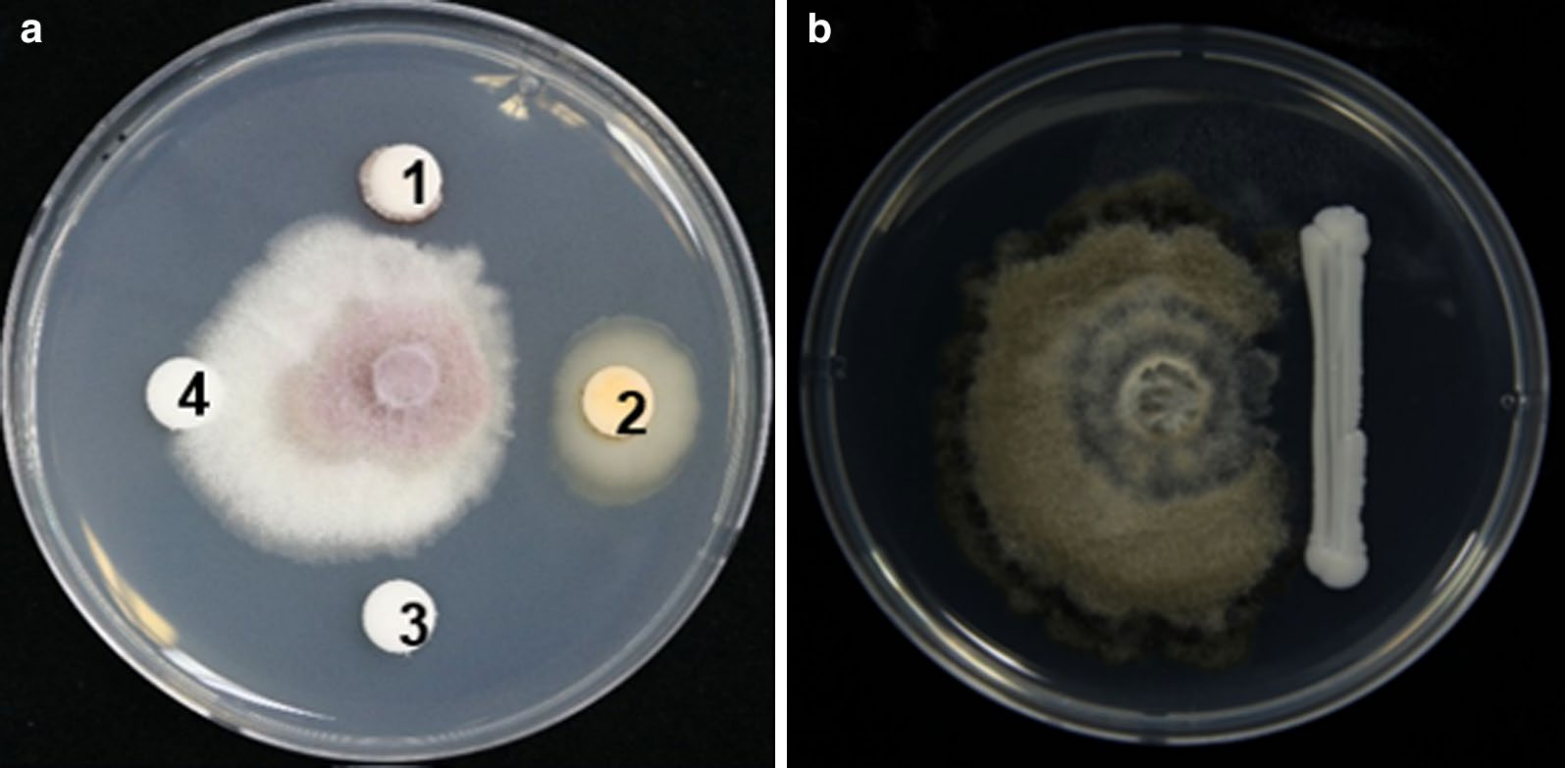
Inhibition test of *M. persimmonesis* on *Fusarium oxysporum* and *Botrytis cineria*

*Metschnikowia persimmonesis* exhibited strong antibacterial activity against *B. cinerea* and *F. oxysporum* (Fig. 9) and was resistant to both antibiotics and streptomycin.

**Fig. 10:**
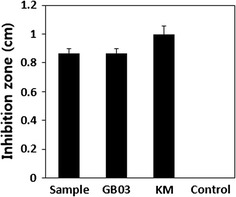
Diameter of growth inhibition zones by *M. persimmonesis* on *Fusarium oxysporum* at 4 days

Thus, the susceptibility of PDA was lower than that of amphotericin and gramicidin S. Studies have showed that a number of members of genus *Metschnikowia* are used in post-harvest control of diseases*. M. fructicola* has been used to manage post-harvest fruit rot (Kurtzman and Droby 2002), whereas *M. pulcherrima* has been used against blue mold in apple (Janisiewicz et al. 2001). As shown by size of zone of inhibition (Fig. 10), the high antimicrobial activity of *M. persimmonesis* may also be explored for biocontrol of post-harvest diseases.

## Abbreviations

KCTC: Korean Collection for Type Cultures
KIOM: Korea institute of oriental medicine
LB: Luria-Bertani
PD: potato dextrose
PDA: potato dextrose agar
PDB: potato dextrose broth
TSA: tryptic soy agar
PCR: polymerase chain reaction
NCBI: National Center for Biotechnology Information
TSB: tryptic soy broth
BLAST: Basic Local Alignment Search Tool
